# Fall 2020 COVID-19 Needs Assessment among New Jersey Secondary School Educational Professionals

**DOI:** 10.3390/ijerph18084083

**Published:** 2021-04-13

**Authors:** Derek G. Shendell, Juhi Aggarwal, Maryanne L. F. Campbell, Lauren N. Gonzalez, Elizabeth Kaplun, Koshy Koshy, Thomas I. Mackie

**Affiliations:** 1 Rutgers School of Public Health (SPH), NJ Safe Schools Program, Rutgers University, Piscataway, NJ 08854, USA; ja880@sph.rutgers.edu (J.A.); mlf159@sph.rutgers.edu (M.L.F.C.); lng45@sph.rutgers.edu (L.N.G.); ek692@sph.rutgers.edu (E.K.); 2 Department of Environmental & Occupational Health & Justice, Rutgers School of Public Health (SPH), Piscataway, NJ 08854, USA; koshyko@sph.rutgers.edu; 3 Department of Epidemiology & Biostatistics, Rutgers School of Public Health (SPH), Piscataway, NJ 08854, USA; 4 Center for Public Health Workforce Development, Rutgers SPH, Somerset, NJ 08873, USA; 5 Department of Health Behavior, Society & Policy, Rutgers School of Public Health (SPH), Piscataway, NJ 08854, USA; thomas.mackie@rutgers.edu

**Keywords:** biological hazards, COVID-19, personal protective equipment (PPE), safety, schools, SARS-CoV-2, teachers, worker health, workplace safety

## Abstract

Secondary or high school (HS) educational professionals expressed concerns about dealing with environmental and occupational health and safety protocols due to COVID-19. Concerns related to fall 2020 school re-opening and getting back into in-person teaching—whether full-time, part-time or some other approved hybrid model—plus ongoing uncertainty with how the state and federal government will be handling matters about mandates for virtual learning, rapid testing, vaccine distribution, etc. These concerns were related to both their experience as educational professionals and genuine interest in personal and student well-being. This study was a cross-sectional online survey in early fall from mid-September–early October 2020. Of a possible maximum participation of 740 New Jersey (NJ) supervisory-level HS teachers and administrators (e.g., department chairs, district and school principals), 100 confirmed unique respondents (13.5%) consented and completed the survey. Of 100 experienced (mean 18 years teaching) participants, 70% responded to the gender identity question (overall, 61% female, 39% male; by NJ region, gender ratios were similar). There were statistically significant differences (using Fischer’s exact test) between NJ regions regarding provision of online counseling and support services for teachers (*p* < 0.001); for resources and equipment for teachers to mediate online learning (*p* = 0.02); for assistive video technology tools (*p* = 0.03) and accessibility to structured online learning and professional development (*p* = 0.002); concerning learning aids to engage students in online instruction, online counseling, and support services for students and their families (*p* = 0.006); appropriate protocol is clean and disinfect areas used by a person with COVID-19 (*p* = 0.002); and, immediately separate staff and students who screen positive for COVID-19 (*p* = 0.03). There were few statistical differences by gender. This study reported what participants wanted regarding the development of future policies then implemented as reopening practices. Data can inform recommendations in NJ and elsewhere at federal, state, and local levels. Data provide new insights and valuable information to inform the consideration of acceptability of various policy measures among HS education professionals.

## 1. Introduction

In the United States (US), secondary or high school (HS) level teachers and administrators or HS educational professionals, are expressing concerns about dealing daily with environmental and occupational safety and health (S&H) due to COVID-19. After moving fully to remote learning in Spring 2020, new concerns emerged in the US, primarily related to the fall 2020 re-opening of schools and getting back to school in-person—whether full-time, part-time or some other approved hybrid model—and the ongoing uncertainty about how the federal government and state government policies would handle mandates for virtual learning, rapid testing, vaccine distribution, etc. These concerns among HS educational professionals have reflected both their expertise and genuine interest in personal and student S&H.

Due to the lack of formal federal guidance, reports with a proposed initial and then revised proposed guidance documents have been published at the national level by non-profits and professional organizations [1,2,3], the National Academies of Science, Engineering and Medicine for K-12 schools [4], and the US Centers for Disease Control and Prevention [5,6]. Academics and practitioners in the US have also weighed in about school and community factors, learning and immunization with rapidly produced peer-reviewed commentaries/opinion pieces in major journals before research and predictive modeling studies were available from the US and other nations [7,8,9,10,11,12,13,14].

To date, primary research on school re-opening has been limited. One study surveyed US parents, guardians, and caregivers near the end of the 2019–2020 school year about fall 2020 K-12 school attendance, regardless of re-opening decisions for the 2020–2021 school year [15]. Another study in the state of Texas conducted a similar survey [16]. Academics and practitioners also documented experiences with school re-opening in other countries in spring or summer 2020 [17,18,19] and conducted global literature reviews to inform layered approaches to reduce virus transmission [20].

In addition, academics have commented on several topics and provided guidance to inform policies and procedures regarding school re-opening. First, re-opening communications for secondary schools must include tailored efforts to adolescents and young adults [21]. Second, re-opening efforts concerning primary school age children must include and consider teachers, staff, parents, caregivers, and guardians [22]. Finally, re-opening plans must consider susceptible, vulnerable sub-groups of K-12 students such as those diagnosed with asthma [2,23] or with various disabilities or special health care needs [4,24].

The situation in the US for the 2020–2021 school year at the state-level was mixed, as some states released formal but typically non-binding guidance—excluding actions taken by statewide executive orders—while other states did not. Overall, re-opening decisions occurred at the local level, such as county boards of education or individual city, town or regional shared school district boards of education [25]. Specifically, in the State of New Jersey (NJ), guidance was released late June 2020 [26] and followed up with updates on virtual learning [27,28] to direct county and local level decisions statewide [29,30].

Given the decentralized decision-making that occurred across the United States, re-opening protocols and procedures require additional information on the front-line perspective of HS professionals who are charged with implementation and are themselves confronted with occupational safety and health concerns. In response to this evidence, this study summarizes results of a multi-part online survey developed in spring-summer 2020 and conducted the first month of the 2020–2021 school year in NJ among secondary school county districts and comprehensive HS programs in career and technical education. Results were divided into self-reported opinions of HS educational professionals in two major areas of local policies and practices: (i) Teaching and learning in the virtual online environment compared to typical in-person classrooms, shops, laboratories, and other school settings; and (ii) HS occupant safety and health.

## 2. Materials and Methods

### 2.1. Study Design and Time Period

This study was a cross-sectional survey created via an iterative process. The NJ Safe Schools Program (NJ SS) received input from multiple state and federal agencies in late Spring-Summer 2020 and then conducted the survey via PsychData (PsychData LLC, State College, PA, USA) for approximately 1 month in early fall, from early September (survey opened right after the Labor Day weekend) through the first weekend of October 2020. The survey development was completed in collaboration with the NJ Department of Education (in particular, the Office of Career Readiness) and the US Centers for Disease Control and Prevention’s National Institute of Occupational Safety and Health (CDC-NIOSH), specifically staff scientists in two different divisions from disciplines of social and behavioral sciences research and injury epidemiology research and practice. These agencies were each provided 2 weeks in July 2020 to comment on proposed questions and answer options, as well as ideas for additional questions. The final survey was agreed on and coded into PsychData in August 2020. NJSS sent the survey by email (an e-newsletter via MyNewsLetterBuilder, JBA Network, Charlotte, NC, USA) on September 17 at midday, September 21 in early afternoon (1st reminder), September 25 in early afternoon (2nd reminder), and September 29 in late morning (3rd and final reminder). Therefore, the e-newsletter readership specifically reached supervisory-level teachers and administrators (e.g., department chairs, district, and school administrators) within New Jersey, referred to as “supervisory NJ high school professionals” hereafter. Teachers and administrators in NJ are eligible to become supervisors of students, and other teachers and school staff, involved in work-based learning (formerly structured learning experiences) including apprenticeships if they are full-time, certified instructors with teaching experience—1–2 or more years depending on career cluster—and complete formal training. The training is a set of four courses with the NJ Safe Schools Program and the Alliance for Young Worker Safety and Health agency partners [31,32]. Please see the Supplementary Materials provided for a copy of the survey.

Since this was framed as a needs assessment survey, this effort was covered by the existing NJ SS human subjects institutional review board approval (IRB Pro no. 021997W0383).

### 2.2. Survey Instrument

The survey contained 22 questions, plus a final section of standard NJ SS six questions about socio-demographics and teaching/administration experience information (but no personal identifiers such as name, email, birthdate/age).

The survey questions were grounded in the understanding that perceptions of supervisory-level educational professionals are critical in the formulation and implementation of organizational responses to new evidence-based understandings of primary and secondary prevention during the COVID-19 pandemic. Consistent with core tenets of implementation science [33], understanding beliefs of supervisory-level educators on the effectiveness and acceptability of practice and policy responses provides information important to the successful implementation of evidence-based policies. In the present case, we considered perceived effectiveness and acceptability of policies that seek to prevent the transmission of COVID-19, facilitate early identification, and prevent or reduce any exposures. Questions were formulated specifically to identify the level of governance perceived accountable for setting a policy and subsequently inquired on the perceived effectiveness of various school initiatives to support a safe return to school. Survey items also inquired on the perceived effectiveness of strategies to promote remote learning. In addition to perceived effectiveness, acceptability by key implementing constituencies is increasingly understood to be critical to generate the buy-in required for successful implementation of evidence-based policies and practice. Accordingly, the survey engaged measures on perceived acceptability of social distancing and measures to promote early detection (e.g., COVID-19 testing and taking forehead (to estimate body) temperature), and to respond to a potential exposure due to an individual having contracted SARS-CoV-2 virus. Please see the Supplementary Materials provided for a copy of the survey.

### 2.3. Study Sample Participants and Demographics

Out of a possible maximum participation of approximately 740 supervisory-level NJ HS educational professionals, 100 confirmed unique respondents (13.5%) consented and completed the survey. They are subsequently referred to as study participants. Some individual questions had either missing data or the participant chose “I do not want to answer” or “Not applicable (to me)”.

Out of 100 participants, 70% responded to the question about gender identity. Overall, the study sample was 61% female and 39% male; no participants self-identified in another way. By the NJ region, gender ratios were similar in North Jersey (63% and 38%) and Central Jersey (69% and 31%). In South Jersey, however, the ratio was closer to 1:1 as only slightly more (53% versus 47%) participants were male.

### 2.4. Data Management and Analyses

Data were downloaded directly from PsychData into Microsoft Excel for data management, including cleaning and recoding, and then analyzed in both Excel and in SAS (v.9.4, Cary, NC, USA). Fisher’s exact tests were used to assess potential differences among participant sub-groups, e.g., statewide by region of NJ or statewide by reported gender (males versus females groups). More specifically, the State of NJ was divided into three regions, with seven counties per region. Region designations were as follows: “North Jersey” included Bergen, Essex, Hudson, Morris, Passaic, Sussex, and Warren counties; “South Jersey” included Atlantic, Burlington, Camden, Cape May, Cumberland, Gloucester, and Salem counties; and, “Central Jersey” included Hunterdon, Mercer, Middlesex, Monmouth, Ocean, Somerset, and Union counties.

## 3. Results and Discussion

### 3.1. Teaching Experience

Of 100 participants, 71% responded to the question about the number of years of teaching experience of participants, overall and within NJ K-12 schools—public, private, and charter combined—through the end of the 2019–2020 school year. Overall, supervisory NJ HS educational professionals working in career and technical education who participated in this study had an average of 18 years of teaching experience (standard deviation (SD): 9.7), with most of the time, about 17 years, within NJ (SD: 8.6); the maximum values were 55 years overall, 43 of which were in NJ. There was little variability by region of NJ or by gender (data not shown). Clearly, the participants in this study sample were an experienced, informed group.

### 3.2. Participant Opinions on Policymaking Questions

We collected data on which level of government agencies the study participants felt, as of early fall 2020, should take the lead in making policies for school re-opening operation procedures “post-COVID-19,” i.e., for the 2020–2021 school year after virtual online learning in March–June 2020. Of 100 participants, 92 (92%) answered this question. Overall, most teachers believed that the state government (41% or 45%) and the school district board of education (32 or 35%), respectively, should set policies on how schools should operate post COVID-19 rather than the local level of government (city/town/borough or county) or the federal/national government (nine or 10%). Only six (7%) of teachers felt as if the local government should take responsibility for the policies. Results are consistent with a recent paper on this issue and government legal powers in the US [34]. This trend was consistent throughout the state; however, in North Jersey, teachers felt as if the federal government was the least responsible party for making school policies compared to the state government and local boards of education. The trend remained consistent between genders; only four (4%) of the participants did not know/did not want to answer the question. There was no statistical difference statewide between the different regions of NJ (*p* = 0.80) or by reported gender (*p* = 0.99).

### 3.3. Participant Practice Opinion Questions

Table 1 describes participant opinions on types of practical initiatives potentially implemented during school re-opening in the 2020–2021 school year. Out of 100 participants, 96% responded to this question. Overall, most participants selected providing cloth face coverings and hand sanitizer to students and personnel, and providing relevant information on the prevention, spread, and containment of COVID-19 to students and personnel, as the most potentially effective methods to make the school buildings and campus facilities safe for students. Nearly half of the participants (47%) reported, however, assessment of school building maintenance needs for ventilation and filtration was the least effective method. This is an interesting finding. This study’s data suggest that participants did not believe sufficient resources were available to obtain the required mechanical ventilation upgrades with advanced filtration required for biological aerosolized agents such as SARS-CoV-2. Compared to face coverings/masks, hand sanitizers, etc. consistently recommended along with social/physical distancing prior to federal reports by US GAO and US EPA 1990s–2021, updated or replaced HVAC systems for adequate if not enhanced ventilation with particle filtration (MERV 8–13 filters) have also been consistently stated, and supplemental portable room air cleaners with HEPA filters would benefit the overall school indoor air and environmental quality [35,36,37,38] and thus school re-opening. However, they did not explicitly focus on aerosolized biological agents, only particles. There were no statistically significant differences statewide between the different regions of NJ or when stratified by the reported gender.

Table 2 describes participant opinions on strategies to support teachers in facilitating remote virtual or online instruction. Out of 100 participants, 84% responded to this question. Teachers reported resources and equipment for teachers to mediate online learning, and online counseling and support services for teachers would be the most effective strategies to support teachers. A recent national presentation on mental health first aid [39] noted the importance of providing mental health and psychosocial supports for teachers and other school personnel to help them improve psychosocial support systems for their students. Moreover, separately, a recent paper on the US 2015–2016 School Survey on Crime and Safety specified HS student mental health services—for both diagnosis and treatment—were limited at present, especially in rural areas compared to urban and suburban areas [40]. In addition, 70% of teachers found that accessibility to structured online learning and professional development are also effective. The NJ Safe Schools Program (see http://www.njsafeschools.org, accessed on 12 April 2021) has already successfully filled this demand with the complete transition of in-person training to online learning (asynchronous plus synchronous “live session” components) for teachers as of late spring 2020 and cohorts of teachers and students starting in winter 2020–2021. There were statistically significant differences between the three NJ regions regarding provision of online counseling and support services for teachers (*p* < 0.001), for resources and equipment for teachers to mediate online learning (*p* = 0.02), for assistive video technology tools (*p* = 0.03), and accessibility to structured online learning and professional development (*p* = 0.002). The other answer options were not statistically different among regions of NJ, and there were no statistical differences between the reported gender.

Table 3 describes the participant opinions on learning aids to engage students in online instruction. Out of 100 participants, 84% responded to this question. Participants reported that assistive video technology tools as well as online counseling and support services for teachers would be the most effective learning aids among the response options presented. Conversely, providing tips to help facilitate interactions between students and teachers, and providing resources and equipment for teachers to mediate online learning, were ranked as the least effective options presented. When stratified by region of NJ only online counseling and support services for students and their families was significant (*p* = 0.006). There were no statistical differences when stratified by gender.

We also collected data on participant opinions on who should be primarily responsible for ensuring students with special healthcare needs are receiving the help they need for both in-class and online instruction. Out of 100 participants, 85% responded to this question. Overall, the clear majority (66% or 78%) of participants believed that either the student’s parents/caregivers/guardians (30% or 35%) and the school district Board of Education (36% or 43%) are the most responsible, not the NJ Department of Education (nine or 11%), while the US Department of Education is the least responsible (seven or 8%). Three participants (4%) answered “other”. This trend is the same, and not statistically significant, between both statewide by regions of NJ (*p* = 0.85) and by the reported gender (*p* = 0.71).

### 3.4. Participant Policy Opinions on Physical or Social Distancing during the COVID-19 Pandemic

Table 4 describes the participant opinions on where social distancing policies are necessary at their schools. Of 100 participants, 96% responded to this question. Nearly every participating secondary school educational professional (between 96–99%) strongly agreed, agreed or felt neutral at the time of this study about social distancing in the following school microenvironments: Classrooms, laboratories, gymnasiums, cafeteria, bathrooms, auditorium, hallways, offices spaces, and on a school bus. On outdoor fields and outdoor playgrounds only 84% of teachers believed that social distancing was important. This might be due to how State of NJ Executive Orders in 2020 promoted the use of available outdoor areas such as recreational open spaces including school activities. Furthermore, 91% of teachers strongly agreed, agreed or felt neutral about how social or physical distancing was important for afterschool activities including allowed interscholastic sports practices and limited competition schedules. This may be in part due to how most K-12 schools have online or hybrid models for the 2020–2021 school year [28,30], and any in-person on-campus teaching is finished in a half-day schedule (excluding allowed outdoor sports activities for practice and competition to date). This might also be because “after school activities” were not defined with detailed examples, therefore, people may have interpreted this as also referring to outdoor activities.

### 3.5. Participant Opinions Pertinent to Safety during the COVID-19 Pandemic

We also collected data to describe the participant opinions on where schools should receive primary funding for personal protective equipment (PPE) and other hygiene-related resources such as cleaning, sanitizing, and disinfecting products. Out of 100 participants, 92% responded to this question. Among those in this study, 34 (37%) of participants believed that funding for PPE and hygiene supplies should come from the federal government and 36 (40%) believed that the state government should be responsible; 12 (13%) believed that the local school district (Board of Education) should be responsible, and six (7%) did not know or did not want to answer. Participants generally seemed to believe that the local government was not responsible (three or 4%). This was interesting, given the fact that the purchase and provision of PPE is the legal responsibility of the employer, i.e., county, regional or local school district [31,41,42]. This trend was consistent among the reported gender (*p* = 0.63). The trend also remained consistent statewide by the region of NJ, with the federal and state government being the most responsible and the local government being the least responsible with no statistical difference (*p* = 0.44).

Data also describe the participant opinions on the practice of separating students and staff who are more susceptible to exposure and vulnerable to contracting the SARS-CoV-2 virus. Out of 100 participants, 87% responded to this question. Overall, 79 (91%) believed that this practice was necessary, and only three (3%) felt this practice was not necessary. Five participants (6%) stated it was somewhat necessary. Responses were consistent statewide and by the reported gender, except in South Jersey where no one found that this practice unnecessary. There were no statistically significant differences between the three NJ regions (*p* = 0.18) or between male and female participants (*p* = 0.93).

### 3.6. Participant Opinions Pertinent to Occupant Health Status during the COVID-19 Pandemic

Furthermore, the data describe the participant opinions on conducting temperature screenings at NJ HS. This study assumed that hand-held forehead thermometers were employed given the State of NJ guidance for re-opening schools [26]. Of 100 participants, 87% responded to this question. Overall, 65 (75%) of participants found that forehead temperature screenings necessary (i.e., very (46% or 53%) or moderately (19% or 22%) necessary), while only six (7%) did not. Sixteen participants (18%) stated it was somewhat necessary. Similarly, by the participant reported gender, 63% of males and 77% of females believed that taking temperature screenings are necessary. Statewide by region of NJ, 65% of teachers in Central Jersey stated taking temperatures was necessary, compared to North Jersey (79%) and South Jersey (71%), whereas only a small number of teachers believed that taking temperatures was not necessary in North Jersey (4%), Central Jersey (10%), and South Jersey (12%). Nevertheless, in part due to the small sample sizes, there were no statistically significant differences statewide between the three NJ regions (*p* = 0.81) or between male and female participants (*p* = 0.65).

The data also describe the participant opinions on how important it is for schools to test students, teachers, and other school personnel for COVID-19 on a bi-weekly basis. It should be noted, however, that the present study did not distinguish between the types of tests currently available [43] for either initial, confirmatory or repeat diagnosis (rapid saliva, swabs for DNA, then PCR analysis) or for prior infection (e.g., antibodies in the blood sample). Out of 100 participants, 87% responded to this question. Overall, at the start of the 2020–2021 school year, teachers reported that bi-weekly COVID-19 testing was necessary and important to them (56% or 64%), but 17 (20%) stated it was not important/not necessary. Fourteen participants (16%) stated it was somewhat necessary. These numbers were not consistent between the different regions of NJ: Only 32% of study participants in Central Jersey did not believe testing is important. Nevertheless, in part due to the sub-group sample sizes, there was no statistical difference between the regions of NJ (*p* = 0.27). There also did not seem to be a large difference between gender; 56% of males and 65% of females found that regular bi-weekly testing for COVID-19 necessary, with no statistically significant difference (*p* = 0.80).

Additionally, the data describe the participant opinions on the appropriate protocol for individuals who contracted COVID-19. Out of 100 participants, 88% responded to this question. Every participating teacher believed that individuals wearing a badge indicating they recently contracted the virus when returning to school was not an appropriate option. Instead, 72 (82%) of participants believed that infected individuals should quarantine at home for 14 days. This remains consistent with official State of NJ guidance [26,27], which remains stricter than the recently updated US CDC guidance [5,6]. The second most common suggestion agreed upon was that information should be provided for at-home learning or teaching (63% or 72%), and the third most common suggestion agreed upon was that individuals should have to receive (at least) two consecutive negative COVID-19 results before returning to school (58% or 66%). It must be noted that this answer option assumed a hybrid model or in-person half-day model was chosen and was approved by the school district and then the NJDOE for the fall 2020 re-opening. These top three answer options selected were consistent statewide between regions of NJ and reported gender; there were no statistical differences when using the Fisher’s exact test.

Table 5 describes the participant opinions on what would be an appropriate protocol for schools to follow regarding COVID-19, i.e., individuals contracting the SARS-CoV-2 virus during the school year. Of 100 participants, 88% responded to this question. Overall, and by region of NJ, the majority (72–89%) of secondary school educational professionals believed that the following should be components of the school-wide protocol: Cleaning and disinfecting areas used by the person with COVID-19; separating staff and students with COVID-19-like symptoms and/or immediately separating them if they tested positive; screening for active COVID-19 among anyone who might have been in contact with the virus; and, notifying both health officials and affected families. Less than but nearly half (47%) of study participants, however, believed that it would be appropriate to close the school temporarily. This is consistent with media reports and updated summaries of NJDOE approvals throughout summer and fall of 2020, i.e., there is a mix of opinions and local-to-county practices about school reopening plans [28,30]. In South Jersey, it must be noted how 100% of participating teachers agreed to clean and disinfect areas used by the person diagnosed with COVID-19. Statewide, there were statistically significant differences between the three regions of NJ among teachers who think the appropriate protocol would be to clean and disinfect areas used by the person with COVID-19 (*p* = 0.002); and, to immediately separate staff and students who screen positive for COVID-19 (*p* = 0.03). Among female participants, immediately separating staff and students with COVID-19 symptoms (*p* = 0.06) as well as staff and students who screen positive for COVID-19 (*p* = 0.01), and notifying health officials (and contact tracers) and affected families (*p* = 0.09) were more important than among male participants.

### 3.7. Limitations and Strengths

The present study holds several limitations worth noting. First, the survey was developed and fielded at a rapid pace to be responsive to the need for immediate information on the perspective of supervisory HS educational professionals working in NJ. Accordingly, not all of the survey measures were validated and psychometric properties were not formally assessed. The NJ Safe Schools Program, however, has routinely used the set of six demographic questions in research and program evaluation settings. Instead, the survey received a review from multiple federal and state expert stakeholders to assess and construct the validity of measures. Second, missing data varied across measures included in the study. This is a function of IRB approval for needs assessment surveys in typical educational settings including online, i.e., consenting participants must have the ability to choose not to answer a question or say “not applicable” or “I do not know”. Third, though we had a statewide sample for this cross-sectional survey-based study, we did not conduct probability (random) sampling. Moreover, of a possible maximum participation of 740 NJ supervisory-level HS teachers and administrators, our final sample size was 100, with a response rate of 13.5%. As a result, the statistics computed were not as robust, i.e., may not represent every secondary career-technical-vocational education school district and comprehensive HS. We developed and conducted this study rapidly in spring-summer 2020 in response to the ongoing crises created by the COVID-19 pandemic. As described in our Methods, we had federal and state input to the survey instrument, even while the formal agency re-opening guidance to schools was limited. Given the fact that supervisory level teachers and administrators (educational professionals) dealt with many things in early fall 2020, this study’s response rate was comparable with or higher than most online cross-sectional surveys conducted without monetary (e.g., e-gift card) or other incentives. Finally, this study, while conducted statewide, was not a probability sample of NJ secondary school educational professionals, and may only be representative of career-technical-vocational HS professionals.

## 4. Conclusions

Overall, when it comes to school policies regarding COVID-19, participating NJ HS educational professionals believed that county, local or regional school district boards of education and the state government were more responsible than federal and local (i.e., city, town) policymakers. This finding was consistent between the gender and regions of NJ. Participants believed that the best strategies for supporting them and their students are to provide resources to both groups to mediate online learning and online counseling for both groups. Participants also believed that their boards of education and the parents are largely responsible for making decisions concerning children with special heath care needs and for the student well-being.

Conversely, participants believed that the State of NJ government and the US Federal/National government should pay for the required personal protective equipment or PPE. Specifically, the initiatives participants believed were more effective are initiatives for primary prevention including providing cloth face coverings and supplies for cleaning, sanitizing, and disinfecting surfaces. Most participants believed, during in-person instruction, that school indoor microenvironments should require social distancing. A majority also said this applies for outdoor areas used for learning, when and where possible.

Regarding COVID-19 policies for practices pertinent to safety and health (S&H), most participants believed that separating students or staff at a greater risk for COVID-19 from those who are at less risk was important. However, fewer participants believed that it was important to have daily screenings (e.g., forehead temperature checks, symptoms surveys). It should be noted how anecdotally, and in many types of media reports, there has been debate on temperature screening accuracy and benefits. Finally, only about two-in-three teachers believed that it was important to test people in the school for COVID-19 on a bi-weekly basis. For each of these participant opinion questions, there was no statistical differences of opinion when stratified by the reported gender and NJ region. When discussing the school protocol, most participants reported that cleaning and disinfecting areas used by people with COVID-19, screening those who have been in contact with COVID-19, and notifying health officials when there are cases were the most important policies to be enacted. Less than half of the teachers believed that the school should be temporarily closed if there are cases of COVID-19.

Data from this study of HS educational professionals in NJ concerning what participants reported wanting in regards to the development of future policies, then implemented as re-opening practices, can inform recommendations for future policies and practices in NJ and elsewhere. These perspectives from those who are charged in mitigating S&H concerns associated with school re-opening for the 2020–2021 school year are critical to federal, state, and local re-opening policies. These data provide new insights and valuable information to inform the consideration of the acceptability of various policy measures among HS education professionals. Findings hold a potential implication to inform both the governance decisions and specific measures taken in updated school re-opening procedures for future emergencies, natural disasters, and infectious disease outbreaks. Indeed, a recent literature review conducted in the European Region of the World Health Organization reported both a lack of government documents and data on policy consensus for schools among high-risk vulnerable sub-populations, as well as for after positive test results [44]. Future research should focus on both the physical S&H of students and HS educational professionals, as well as their mental health.

## Figures and Tables

**Table 1:** Initiatives school systems can take to get back to school safely. Data presented for the overall study sample. Fisher’s exact tests were used to document how, for each initiative, there were no statistically significant differences statewide by regions of New Jersey (NJ) or when stratified by the reported gender.

Total *N* = 96	Least/Less Effective		Medium Effectiveness		Most/More Effective	
	*N*	%	*N*	%	*N*	%
Provide cloth face coverings and hand sanitizers to students/personnel	31	32.3	26	27.1	39	40.6
Provide sanitation/disinfecting supplies to students/personnel	31	32.3	31	32.3	34	35.4
Provide relevant information on the prevention, spread, and containment of COVID-19 to students/personnel	37	38.5	26	27.1	33	34.3
Establish school-wide procedures for students/teachers who feel unwell	18	18.8	49	51.0	29	30.2
Develop school-wide emergency plans in case of exposure	30	31.2	37	38.5	29	30.2
Assess school building maintenance needs for ventilation and filtration	45	46.8	23	24.0	28	29.2

**Table 2:** Strategies to support teachers in facilitating remote/online instruction. Data presented for the overall study sample. Note: *p*-values were determined by Fisher’s exact test.

Total *N* = 84	Least/Less Effective		Medium Effectiveness		Most/More Effective		*p*-Value Stratified by Gender	*p*-Value Stratified by NJ Region
	*N*	%	*N*	%	*N*	%		
Assistive video technology tools	39	46.4	18	21.4	27	32.1	0.73	0.03 **
Accessibility to structured online learning and professional development	26	31.0	29	34.5	29	34.5	0.95	0.002 ***
Online counseling and support services for teachers	37	44.0	10	11.9	37	44.0	0.19	0.005 ***
Provide tips to help facilitate interactions between students and teachers	34	40.5	16	19.0	34	40.5	0.76	0.21
Provide resources and equipment for teachers to mediate online learning	33	39.3	11	13.1	40	47.6	0.56	0.02 **

* *p*-value ≤ 0.10, ** *p*-value ≤ 0.05, *** *p*-value ≤ 0.01.

**Table 3:** Learning aids to engage students in remote/online instruction. Note: P-values were determined by Fisher’s exact test.

Total *N* = 84	Survey Question Response *N*	Least/Less Effective		Medium Effectiveness		Most/More Effective		*p*-Value Stratified by Gender	*p*-Value Stratified by NJ Region
		*N*	%	*N*	%	*N*	%		
Assistive video technology tools	82	35	42.7	14	17.1	33	39.3	0.69	0.83
Accessibility to structured online learning and tutoring resources	82	19	22.6	23	28.0	40	48.8	0.76	0.74
Online counseling and support services for students and their families	83	29	34.9	16	19.3	38	45.8	0.68	0.006 **
Provide tips to help facilitate interactions between students and teachers	83	44	53.0	18	21.7	21	25.3	0.49	0.70
Provide resources and equipment to student’s parents/caregivers to mediate online learning	84	38	45.2	12	14.3	34	40.5	0.41	0.27

* *p*-value ≤ 0.10, ** *p*-value ≤ 0.05, *** *p*-value ≤ 0.01.

**Table 4:** Study participant level of agreement for each school setting with the following statement: “Social distancing policies are necessary in… [microenvironment listed] …”.

Total *N* = 90	Strongly Agree/Agree		Neutral		Disagree/Strongly Disagree	
Microenvironment	*N*	%	*N*	%	*N*	%
Classrooms	85	94.4	3	3.3	2	2.2
Laboratories	81	90.0	7	7.8	2	2.2
Gymnasium	84	93.3	4	4.4	2	2.2
Cafeteria	84	93.3	5	5.6	1	1.1
Bathrooms	81	90.0	6	6.7	3	3.3
Auditorium	85	94.4	4	4.4	1	1.1
Hallways	79	87.8	7	7.8	4	4.3
Office spaces	76	84.4	10	11.1	4	4.3
Busses	82	91.1	4	4.4	4	4.3
Outdoor field/playground	61	67.8	15	16.7	14	15.6
After-school activities	77	85.6	5	5.6	8	8.9

**Table 5:** Which of the following do you feel would be an appropriate protocol for the school? Stratified by regions of NJ and reported gender. Note: *p*-values were determined by Fisher’s exact test.

Total *N* = 88	Overall (*N* = 88)		North Jersey (*n* = 24)		Central Jersey (*n* = 31)		South Jersey (*n* = 17)		*p*-Value	Males (*n* = 27)		Females (*n* = 43)		*p*-Value
	*N*	%	*N*	%	*N*	%	*N*	%		*N*	%	*N*	%	
Clean and disinfect areas used by the person with COVID-19	78	88.6	16	66.7	31	100	17	100	0.0002 ***	23	85.2	39	90.7	0.7
Immediately separate staff and students with COVID-19 symptoms	63	71.6	16	66.7	22	71.0	14	82.4	0.53	16	59.3	35	81.4	0.06 *
Screen for active COVID-19 among anyone who might have been in contact	73	83.0	18	75.0	25	80.6	16	94.1	0.31	21	77.8	36	83.7	0.54
Immediately separate staff and students who screen positive for COVID-19	63	71.6	15	62.5	19	61.3	16	94.1	0.03 **	14	51.9	35	81.4	0.01 ***
Notify health officials (and contact tracers) and affected families	75	85.2	19	79.2	26	83.9	16	94.1	0.46	20	74.1	39	90.7	0.09 *
Temporarily close the school	41	46.6	11	45.8	18	58.1	6	35.3	0.29	10	37.0	25	58.1	0.14
None of above	0	0	0	0	0	0	0	0	-----	0	0	0	0	-----
Other	7	8.0	0	0	2	6.4	3	17.6	0.09 *	2	7.4	3	7.0	1.00

* *p*-value ≤ 0.10, ** *p*-value ≤ 0.05, *** *p*-value ≤ 0.01.

## Data Availability

This study’s data are secured on computers per the IRB approved stewardship of the New Jersey Safe Schools Program and/or are publicly available from the New Jersey Department of Education. Datasets used and analyzed during the current study are available from the corresponding author on a reasonable request.

## Supplementary Materials

This is available online at https://www.mdpi.com/article/10.3390/ijerph18084083/s1, Figure S1: Copy of Needs Assessment Survey Questions.
