# Supplementary material for: Fall 2020 COVID-19 Needs Assessment among New Jersey Secondary School Educational Professionals
Source: Int J Environ Res Public Health. 2021 Apr 13;18(8):4083. doi: 10.3390/ijerph18084083 (PMC8070613; doi:10.3390/ijerph18084083)
Supplement: Supplementary file 1 [file ijerph-18-04083-s001.pdf]

Supplemental Information

Getting Back to School Needs Assessment

1. Please rank order the initiatives school systems can take to get back to school safely from least effective [1] to most effective [6].
    a. Provide cloth face coverings and hand sanitizer to students and school personnel
    b. Provide sanitation/disinfecting supplies to students and school personnel
    c. Provide relevant information on the prevention, spread, and containment of COVID-19 to students and school personnel
    d. Establish schoolwide procedures for students and teachers who feel unwell
    e. Develop schoolwide emergency plans in case of exposure
    f. Assess school building maintenance needs for ventilation and filtration

2. In your opinion, which level of government should set the policies governing how schools operate post-COVID-19? Check only one.
    a. School District Board of Education
    b. Local government (e.g., town, city)
    c. State government
    d. Federal government
    e. I do not know.
    f. I do not want to answer.

3. In your opinion, where should your school receive primary funding for PPE and other resources such as sanitation and disinfecting products come from? Check only one.
    a. School District Board of Education
    b. Local government (e.g., town, city)
    c. State government
    d. National/Federal government
    e. I do not know.
    f. I do not want to answer.

4. Please choose your level of agreement with the following statement for each of the following school settings: Social distancing policies are necessary (Strongly agree, agree, neither agree nor disagree, disagree, strongly disagree [5-point Likert scale])
    a. Classrooms
    b. Laboratories
    c. Gymnasium
    d. Cafeteria
    e. Bathrooms
    f. Auditorium
    g. Hallways
    h. Office spaces
    i. Busses
    j. Outdoor field/playground
    k. After-school activities
    l. Other (please write in your thoughts): _________

5. If an individual at the school contracts COVID-19 at any time during the school year, which of the following do you feel would be an appropriate protocol **for the individual who contracted COVID-19**? Check all that apply.
    a. Provide the individual with information for at-home learning or teaching
    b. Have individual quarantine at home for 14 days
    c. Have individual obtain two negative COVID-19 tests before returning to school
    d. Have individual wear a badge indicating they recently contracted the virus when returning to school
    e. None of the above
    f. Other (please write in your thoughts): __________

6. If an individual contracts COVID-19 at any time during the school year, which of the following do you feel would be an appropriate protocol **for the school**? Check all that apply.
    a. Clean and disinfect areas used by the person with COVID-19
    b. Immediately separate staff and students with COVID-19 symptoms
    c. Screen for active COVID-19 among anyone who might have been in contact
    d. Immediately separate staff and students who screen positive for COVID-19
    e. Notify health officials (and contact tracers) and affected families
    f. Temporarily close the school
    g. None of the above
    h. Other (please write in your thoughts): ___________

7. In your opinion, how necessary is separating students and staff at greater risk of more serious illness due to COVID-19 from those at less risk? Check only one.
    a. Very necessary
    b. Moderately necessary
    c. Somewhat necessary
    d. Not necessary

8. In your opinion, how necessary are temperature screenings at school? Check only one.
    a. Very necessary
    b. Moderately necessary
    c. Somewhat necessary
    d. Not necessary

9. In your opinion, how important is it for schools to test students, teachers, and other school personnel for COVID-19 on a bi-weekly basis? Check only one.
    a. Very important
    b. Moderately important
    c. Somewhat important
    d. Not important

10. Who do you think should be primarily responsible for ensuring students with special healthcare needs are receiving the help they need for both in-class and online instruction? Check only one.
    a. Parents/caregivers
    b. School District Board of Education
    c. NJ Department of Education
    d. United States Department of Education
    e. Other State of NJ agency (please write in your thoughts): ____________________

11. Please rank order the following strategies to support teachers in facilitating remote/online instruction from least useful [1] to most useful [5].
    a. Assistive video technology tools
    b. Accessibility to structured online learning and professional development
    c. Online counseling and support services for teachers
    d. Provide tips to help facilitate interactions between students and teachers
    e. Provide resources and equipment for teachers to mediate online learning

12. Please rank order the following learning aids to engage students in remote/online instruction from least useful [1] to most useful [5].
    a. Assistive video technology tools
    b. Accessibility to structured online learning and tutoring resources
    c. Online counseling and support services for students and their families
    d. Provide tips to help facilitate interactions between students and teachers
    e. Provide resources and equipment to student's parents/caregivers to mediate online learning

[The following questions will be on final page/screen.]

Demographic information

13. County
14. School district
15. School name
16. Gender
    a. Female
    b. Male
    c. Other
    d. Prefer not to answer
17. Number of years teaching in NJ
18. Number of years teaching overall
